# Survival of Cancer Stem Cells under Hypoxia and Serum Depletion via Decrease in PP2A Activity and Activation of p38-MAPKAPK2-Hsp27

**DOI:** 10.1371/journal.pone.0049605

**Published:** 2012-11-20

**Authors:** Shih-Pei Lin, Yi-Ting Lee, Jir-You Wang, Stephanie A. Miller, Shih-Hwa Chiou, Mien-Chie Hung, Shih-Chieh Hung

**Affiliations:** 1 Institute of Clinical Medicine, National Yang-Ming University, Taipei, Taiwan; 2 Institute of Pharmacology, National Yang-Ming University, Taipei, Taiwan; 3 Stem Cell Laboratory, Department of Medical Research and Education, Taipei Veterans General Hospital, Taipei, Taiwan; 4 Orthopaedics and Traumatology, Taipei Veterans General Hospital, Taipei, Taiwan; 5 Department of Molecular and Cellular Oncology, The University of Texas M.D. Anderson Cancer Center, Houston, Texas, United States of America; 6 Graduate Institute of Cancer Biology, College of Medicine, and Center for Molecular Medicine, China Medical University and Hospital, Taichung, Taiwan; University Health Network, Canada

## Abstract

Hypoxia and serum depletion are common features of solid tumors that occur upon antiangiogenesis, irradiation and chemotherapy across a wide variety of malignancies. Here we show that tumor cells expressing CD133, a marker for colorectal cancer initiating or stem cells, are enriched and survive under hypoxia and serum depletion conditions, whereas CD133− cells undergo apoptosis. CD133+ tumor cells increase cancer stem cell and epithelial-mesenchymal transition properties. Moreover, via screening a panel of tyrosine and serine/threonine kinase pathways, we identified Hsp27 is constitutively activated in CD133+ cells rather than CD133− cell under hypoxia and serum depletion conditions. However, there was no difference in Hsp27 activation between CD133+ and CD133− cells under normal growth condition. Hsp27 activation, which was mediated by the p38MAPK-MAPKAPK2-Hsp27 pathway, is required for CD133+ cells to inhibit caspase 9 and 3 cleavage. In addition, inhibition of Hsp27 signaling sensitizes CD133+ cells to hypoxia and serum depletion -induced apoptosis. Moreover, the antiapoptotic pathway is also activated in spheroid culture-enriched CD133+ cancer stem cells from a variety of solid tumor cells including lung, brain and oral cancer, suggesting it is a common pathway activated in cancer stem cells from multiple tumor types. Thus, activation of PP2A or inactivation of the p38MAPK-MAPKAPK2-Hsp27 pathway may develop new strategies for cancer therapy by suppression of their TIC population.

## Introduction

The heterogeneous phenotypical and molecular traits of human cancers are a function of their tumor initiation or cancer stems cells (TICs) content [1]. The CD133+ population of cells account for about 2.5% of colon cancer tumors cells [2], and previous studies have shown that this population of cells contains a number of undifferentiated tumorigenic TICs [2], [3]. They showed that subcutaneous injection of CD133+ colorectal cancer cells, but not CD133− cells, were able to readily reproduce the original tumor in immunodeficient mice. Deregulation of TIC self-renewal is a likely requirement for the development of cancer [4], [5], [6], and survival of TICs may be responsible for the resistance to cancer therapies and recurrence of tumors [7]. The underlying mechanisms of TICs survival from antitumor therapies are largely unknown. However, an increase in ABC transporters [8], active DNA-repair capacity [7], and resistance to apoptosis by production of cytokines or activation of specific pathways have been reported. Thus, identifying the signaling pathways of the survival and self-renewal properties of TICs has recently attracted a great deal of attention, owing to the promise of a novel cellular target for the treatment of cancers.

Heat shock proteins (Hsps) play an essential role as molecular chaperones by assisting the correct folding of stress-accumulated misfolded proteins, and directly interacting with various components of the tightly regulated programmed cell death machinery. Among them, the Hsp27 basal level is usually high in cells or tissues from a wide range of tumors [9]. Additionally, it has been demonstrated that Hsp27 enhances resistance to chemotherapy with cisplatin and doxorubicin, and increases the tumoriogenic potential of rat colon cancer cells [10].

Hypoxia and serum depletion are common features of solid tumors that occur upon antiangiogenesis, irradiation and chemotherapy across a wide variety of malignancies [11], [12]. Hypoxia and anemia (which contributes to tumor hypoxia) can lead to ionizing radiation and chemotherapy resistance by depriving tumor cells of the oxygen essential for the cytotoxic activities of these agents [13], [14]. Hypoxia may also reduce tumor sensitivity to radiation therapy and chemotherapy through one or more indirect mechanisms that include proteomic and genomic changes. These effects, in turn, can lead to increased invasiveness and metastatic potential, loss of apoptosis, and chaotic angiogenesis, thereby further increasing treatment resistance. However the response of tumor cells to hypoxia and serum depletion and the underlying mechanism mediates this response remain to be clarified. In the current study, we demonstrated that most of colon cancer cells undergo apoptosis upon exposure to serum depletion and hypoxia, and the CD133+ population of colon cancer cells is more resistant to apoptosis through the constitutive activation of an anti-apoptotic signaling pathway involving Hsp27. Moreover, we show that TICs can be sensitized to undergo apoptosis and cell death through the inactivation of the Hsp27 pathway.

## Materials and Methods

### Cell Culture and Hypoxic Conditions

The human colorectal cancer cell line HT-29 was obtained from the American Type Culture Collection (ATCC). The CCS and HCW primary culture cells were gifted by Dr. Wen K. Yang (Laboratory of Cell/Gene Therapy, China Medical University Hospital, Taichung, Taiwan). The CCS cells were isolated from a primary tumor of a female with Duke C_3_ colon adenocarcinoma. The HCW cells were isolated from the liver metastasis sample of a male with colon adenocarcinoma. All of the cells were grown in DMEM (Gibco, Grand Island, NY,) containing 10 units/mL penicillin, 10 µg/mL streptomycin, 2 mmol/L glutamine, and 10% fetal bovine serum (FBS; Gibco), in 37°C humidified atmosphere with 5% CO_2_. For hypoxic conditions, cells were cultured in a gas mixture composed of 94% N_2_, 5% CO_2_, and 1% O_2_, which was maintained using an incubator with two air sensors, one for CO_2_ and the other for O_2_; the O_2_ concentration was achieved and maintained using delivery of nitrogen gas (N_2_). If O_2_ percentage rises above the desired level, N_2_ gas was automatically injected into the system to displace the excess O_2_. For spheroid culture, cells were resuspended in serum-free DMEM/F12 medium supplemented with N2 supplement, recombinant human EGF (20 ng/mL; PeproTech), and FGF (10 ng/mL; PeproTech), and plated at a density of 10^4^ cells/well in a 6-well Ultra-Low Attachment Microplate (Corning, Lowell, MA).

### Assays for Apoptosis

Apoptosis was assayed with a cellular dye that detects membrane alterations (phosphatidylserine flip) and stains apoptotic cells (APOPercentage; Accurate Chemical & Scientific Corporation, New York, NY). Stained cells were assayed using a Spectramax 250 microplate reader (Molecular Devices, Sunnyvale, CA). Alternatively, cells were harvested by trypsin treatment, stained with TUNEL assay (In Situ Cell Death Detection Kit, Roche), and visualized using fluorescence microscope. For the detection of the cleaved forms of caspase 3 and 9, protein lysates were prepared and a western blot was performed with the primary antibodies purchased from Cell Signaling Technologies (Beverly, MA).

### Cell Migration and Invasion Assay

For migration assay, 4×10^4^ cells, suspended in 200 µl of DMEM supplemented with 0.5% FBS, were seeded in the upper well of 24-well transwell that contained a filter with 8 µm pores (Corning, costar, USA). In the lower well, 600 µl of DMEM supplemented with 15% FBS was added. Cell were incubated at 37°C for 24 h. Cells those retained in the upper well were removed by swab and those migrated to the opposite side of the filter were stained with DAPI and counted under a fluorescence microscope at 200× magnification. The invasive capability of the cells was determined with Matrigel (Becton Dickinson)-coated polycarbonate filters with 8 µm pores of 24-well transwell. Cell seeding density, culture medium, culture period, and evaluation method were the same with the migration assay.

### Real-time RT-PCR

The method of real-time RT-PCR was performed as described [15]. Briefly, total RNA (1 µg) of each sample was reversely transcribed in 20 µL using 0.5 µg of oligo dT and 200 U Superscript III RT (Invitrogen, Carlsbad, CA). The amplification was carried out in a total volume of 20 µL containing 0.5 µM of each primer, 4 mM MgCl_2_, 2 µL of LightCycler™–FastStart DNA Master SYBR green I (Roche Molecular Systems, Alameda, CA) and 2 µL of 110 diluted cDNA. PCR reactions were prepared in duplicate and heated to 95°C for 10 min followed by 40 cycles of denaturation at 95°C for 15 sec, annealing at 60°C for 1 min, and extension at 72°C for 20 sec. Standard curves (cycle threshold values versus template concentration) were prepared for each target gene and for the endogenous reference (GAPDH) in each sample. The quantification of the unknown samples was performed by the LightCycler Relative Quantification Software version 3.3 (Roche).

### Flow Cytometric Analysis, Sorting and MACS-separation

Suspensions of cancer cells lifted with EDTA were washed with phosphate-buffered saline (PBS) and incubated for 30 min at 4°C with FITC- or PE-conjugated monoclonal antibodies to human CD markers in 50 µL of washing buffer (PBS, 2% FBS). After incubation, cells with bound antibodies were washed twice with washing buffer and fixed in 1% paraformaldehyde (in PBS). Mouse isotype antibodies served as respective controls. Cells were analyzed using a FACScan flow cytometer running CellQuest software (Becton Dickinson, San Jose, CA). Cells used for sorting was prepared as the method for flow cytometry with all the reagents aseptic and without any fixation. Magnetic cell separation (MACS) of CD133+ cells was performed according to the manufacture’s instructions (http://www.miltenyibiotec.com/en/NN_21_MACS_Cell_Separation.aspx). After incubation with immunomagnetic CD133-microbeads (Miltenyi Biotec, Bergisch-Gladbach, Germany) for 30 min at 4°C, cells were washed in PBS plus 0.5% BSA plus 2 mM EDTA, filtered through a 40-µm cell strainer, and run over a magnetic cell separation device (Auto-Macs; Miltenyi Biotec) for positive selection of CD133+ cells.

### Western Blot Analysis

Cell extracts were prepared with M-PER (Pierce, Rockford, IL) plus protease inhibitor cocktail (Halt™; Pierce) and protein concentrations were determined using the BCA assay (Pierce). Aliquots of protein lysates were separated on SDS–10% polyacrylamide gels and transferred to PVDF membrane filters, which were blocked with 5% blotting grade milk (Bio-Rad, Hercules, CA) in TBST (20 mM Tris-HCl [pH 7.6], 137 mM NaCl, 1% Tween 20). Membranes were then probed with the indicated primary antibodies, reacted with corresponding secondary antibodies, and detected using a chemiluminescence assay (Millipore, Billerica, MA). Membranes were exposed to X-ray film to visualize the bands (Amersham Pharmacia Biotech, Piscataway, NJ). Antibodies against pPP2A (#12615; Santa cruz), pP38 (#9216; Cell Signaling), pMAPKAPK2 (#3007; Cell Signaling), pHsp27 (AF2314, R&D), β-tubulin (#05-661; Millipore), E-cadherin (#4065; Cell Signaling), N-cadherin (#4061; Cell Signaling), vimentin (MAB3400; Millipore), fibronectin (sc-8422; Santa Cruz), pJak2 (#3771; Cell Signaling) and pc-Src at Tyr416 (#2101; Cell Signaling) were used. PP2 and PP3 were purchased from Calbiochem.

### Immunofluorescence and Immunohistochemical Staining

For immunofluorescence, spheres were treated with blocking buffer, incubated with mouse or rabbit antibodies against human CD133 (AC133 mouse IgG, Miltenyi), CK 20 (Ks20.8 mouse IgG2a, GeneTex, San Antonio, TX), CDX2 (Chemicon, Temecula, CA), and β-catenin (H-102, Santa Cruz Biotechnology; Santa Cruz, CA), at appropriate dilutions overnight at 4°C, washed extensively with PBS, and reacted with corresponding fluorescein isothiocyanate (FITC)-conjugated secondary antibodies. Immunofluorescence was observed with fluorescence microscope. For immunohistochemical staining, paraffin-embedded tumor sections were deparaffinized, rehydrated and antigen retrieved by placing sections in Declere working solution (Cell Marque, Austin, TX) in a microwave oven for 20 min. Endogenous peroxidase activity was blocked by 3% hydrogen peroxide. Residual enzymatic activity was removed by washes in PBS, and non-specific staining was blocked with Ultra V Block for 5 min (Thermo Fisher Scientific, Fremont, CA). Then the sections were reacted with first antibodies overnight at 4°C, washed extensively with PBS, and reacted with corresponding biotinylated secondary antibodies (Vector Laboratories, Burlingame, CA) for 15 min at room temperature and treated with streptavidin-peroxidase (LSAB Kit; Dako, Carpinteria, CA), followed by diaminobenzidine staining. Counterstaining was performed with Mayer’s hematoxylin.

### Screening Detection of Phosphorylation of RTKs and Family of MAPK

Membranes from the Human Phospho-Receptor Tyrosine Kinase Array Kit (Catalog Number ARY001; R&D Systems, Minneapolis, MN) and the Human Phospho-MAPK Array Kit (Catalog Number ARY002; R&D Systems) were blocked with Array Buffer I for 1 h. Diluted protein lysates in Array Buffer I were incubate overnight at 2–8°C (or 2 h at room temperature). The membranes were washed three times with 1×Wash Buffer at room temperature, and incubated with freshly diluted Detection Antibodies for 2 h at room temperature. The membranes were washed and detected using a chemiluminescence assay. Membranes were exposed to X-ray film to visualize the dots.

### Lentiviral Vector Production and Cell Infection

The expression plasmids and the bacteria clones for Hsp27 shRNA (shRNA1: TRCN0000008753, shRNA1: TRCN0000011466) were provided by the RNAi core of National Science Council in Taiwan. The shRNA lentiviral vectors were produced by transfection of 293FT cells using Lipofectamine 2000 (LF2000; Invitrogen, Carlsbad, CA). Supernatants were collected 48 h after transfection and then were filtered. Subconfluent cells were infected with lentivirus in the presence of 8 µg/mL polybrene (Sigma-Aldrich). At 24 h post-infection, we removed medium and replaced with fresh growth medium containing puromycin (4 µg/ml) and selected for infected cells for 48 h.

### PP2A Phosphatase Activity Assay

PP2A activity was assayed using the phosphatase kit V2460 (Promega, Madison, WI). Briefly, cells separated by MACS or enriched for TICs were lysed in phosphatase storage buffer, followed by removal of endogenous phosphate using spin columns provided by serine/threonine phosphatase assay system. 1 μg protein samples in triplicates were incubated with phosphopeptide in PP2A reaction buffer for 2 h at room temperature. After washing, the samples were colorized by dye mixture for 15 min followed by stopping the reaction, and the OD values at 600 nm were measured. The data were normalized with control cells as 100%.

### Statistical Analysis

All values are expressed as mean ± SD. Comparisons between two groups were analyzed by Student t-test. A value of p<0.05 was considered statistically significant.

## Results

### Hypoxia and Serum Depletion Increase the Population of CD133+ cells

**Figure 1:**
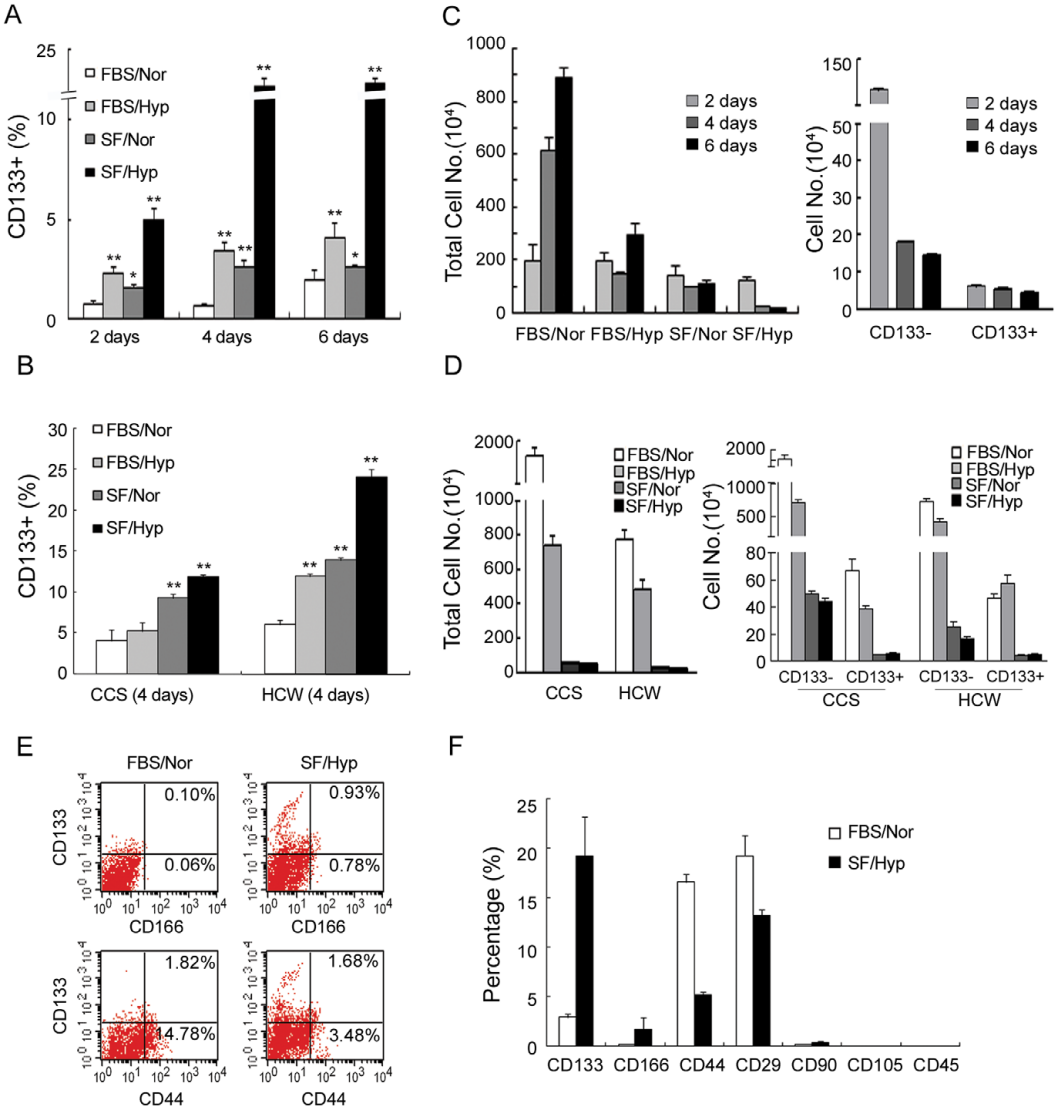
CD133+ cells increase in percentage under hypoxia and serum depletion. (A) HT-29, (B) CCS and HCW cells were seeded with normal growth medium (FBS) under normoxia (Nor). Cells were exposed to serum depletion (SF), hypoxia (Hyp), hypoxia and serum depletion, or, as a control, under the same condition, and 2, 4 or 6 days later, the cells were harvested for assaying CD133 percentage by flow cytometry. (C left panel) Total cell numbers for each condition and (C right panel) cell number of CD133− and CD133+ of HT-29 cells at indicated period under hypoxia and serum depletion were measured. (D left panel) Total cell number and (D right panel) numbers of CD133+ and CD133− cells of CCS and HCW cells at 4 days under each condition. (E and F) Flow cytometry for surface protein profiles of cells cultured under control, or under hypoxia and serum depletion for 4 days.

Exposure of colon cancer cells to both hypoxia and serum free medium resulted in a large number of cell death, suggesting most of the tumor cells did not survive under these conditions. However, we found that the percentage of CD133+ cells of HT-29 colon cancer cells increased a few fold under hypoxic conditions and also with serum free medium, as compared to normoxic conditions. However, under both hypoxia and serum free medium conditions, the CD133+ population was increased up to almost 30-fold (Figure 1A). The increase in the percentage of CD133+ cells was seen after 2 days and even more dramatically on day 4 or 6. This increase in CD133+ cells was also seen in the CCS and HCW primary culture colon cancer cells (Fig. 1B). The increase in percent of CD133+ cells, however, was not due to an increase in the total number of CD133+ cells, as the actual number of CD133+ cells decreased slightly along with the increase of culture under hypoxia and serum free medium conditions (Figure 1C) or compared to the number of CD133+ cells under serum-contained medium or/and normoxia conditions (Figure 1D). Since the CD44+ and CD166+ cells are also considered as the TICs in colorectal cancer [16], we also asked whether these markers were associated with CD133 and affected by hypoxia and serum depletion. The majority of CD133+ cells were found to be negative for both CD44 and CD166, however, with hypoxia and serum depletion we observed an increase in CD166+ cells, but not CD44+ and the population of CD166+ was much smaller than that of CD133+ (Figure 1E), suggesting that the majority of the CD133+ population is distinct from the CD166+/CD44+ cells. As expected, CD133+ cell were stained negative for CD29, CD90 and CD105, markers for mesenchymal stem cells and CD45, marker of haematopoietic cells (Figure 1F). No cells were stained positive for CD105 and CD45, and none of these markers were increased under serum depletion and hypoxia conditions (Figure 1F).

### CD133+ cells Possess Properties of Colorectal TICs

**Figure 2:**
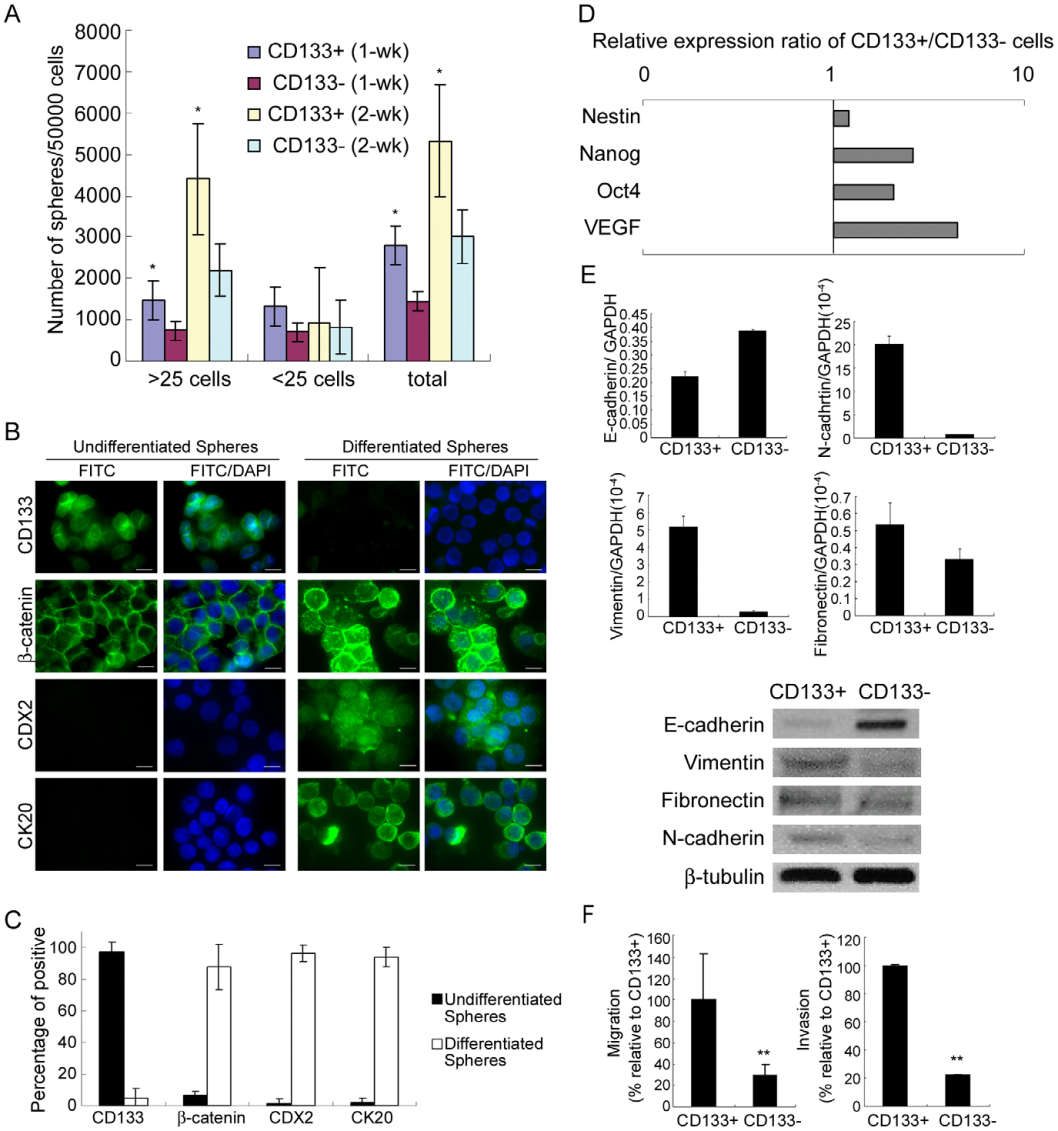
CD133+ cells form undifferentiated spheres and increase in embryonic gene expression and markers for EMT. (A)Sphere formation capacity of CD133+ and CD133− cells. CD133+ and CD133− cells, separated by MACS after 4 days cultured under hypoxia and serum depletion, were cultured under serum free conditions in the presence of EGF (10 ng/mL) and FGF2 (10 ng/mL). Sphere formation was calculated at 2 weeks (>25 or <25 indicates the cell number of each sphere.). Error bars represent standard deviation. (*p<0.05 compared with CD133−cells as determined by the Student’s t test.) (B) CD133+ cells after MACS separation at day 4 of exposure to hypoxia and serum depletion, cultured under serum free condition in the presence of EGF (10 ng/mL) and FGF2 (10 ng/mL), formed spheres at 2 weeks. Immunofluorescence shows that these spheres are undifferentiated spheres (Undifferentiated), which are positive for CD133, and negative for nuclear β-catenin, CDX2 and CK20. The spheres were then seeded into matrigel and exposed to serum-containing condition for another 2 weeks. Immunofluorescence shows that these spheres are differentiated (Differentiated), which decrease in CD133 expression, increase in the expression of nuclear β-catenin, CDX2 and CK20. Scale bar, 20 µm (C) Percentages of cells positive for CD133, nuclear β-catenin, CDX2 and CK20 were counted. (D) and (E upper two panels) Quantitative RT-PCR for mRNA levels, (E lower panel) immunoblot analysis. (F) cell migration and invasion assay for CD133+ and CD133− cells after MACS separation at day 4 of exposure to hypoxia and serum depletion. Data of HT-29 cells are shown as representative results.

To further address whether the CD133+ population are bona fide colorectal TICs we used the following properties associated with colorectal TICs in the literature to characterize the CD133+ population. Since one essential characteristic ability of TICs is to form undifferentiated spheres (spheroids) [2], we asked whether the CD133+ cells isolated from hypoxia and serum depletion possess the ability. We found that CD133+ cells were able to form spheres more readily than CD133− cells when cultured in serum free medium supplemented with EGF and FGF2 (Figure 2A). As expected, the cells in the spheres were positive for CD133, but negative for differentiated markers of colorectal tumors, i.e. nuclear β-catenin (activated β-catenin), cytokeratin 20 (CK20) and the caudal type homeobox transcription factor 2 (CDX2) (Figure 2B and 2C), indicating these spheres were undifferentiated spheres. To investigate if the enriched CD133+ population in the spheres possesses the ability to form differentiated markers of colorectal tumors, CD133+ spheres were seeded into matrigel and exposed to serum-containing medium. After culturing in these conditions for 2 weeks, they started to form differentiated spheres that were decreased in CD133 staining, positive for nuclear β-catenin, CK20 and CDX2 (Figure 2B and 2C). Importantly, for CD133+ cells, injection of less than 1.000 cells was sufficient to form tumors when transplanted into immunodeficient mice, but even with 100,000 cells, CD133− were not able to form tumors (Figure S1A). To be expected, tumors formed by CD133+ cells, similar to tumors formed by bulk cells, were positively stained for colorectal cancer markers (Figure S1B). Moreover, xenograft tumors formed by CD133+ also contained CD133+ and CD133− cells (Figure S1B), and the CD133+ population isolated from xenograft tumors formed a population of cells mixed with CD133+ and CD133− cells *ex vivo*, while the CD133− population still gave rise to CD133− cells (Figure S1C). These data demonstrated CD133+ cells formed xenograft tumor and gave rise to both of CD133+ and CD133− cells, suggesting these CD133+ cells possessed the properties of TICs. In addition, quantitative RT-PCR demonstrated that CD133+ cells had an increase of the expression of several stem cell related genes including, Oct4, Nanog, Nestin, KLF4, Notch1, Notch 2 and Notch 3, c-myc, Gil1, Wnt5a and VEGF (Figure 2D and Figure S2). Oct4 and Nanog are exclusively expressed in embryonic stem cells [17], Nestin is expressed in pluripotent neural stem cells [18], while KLF4, c-myc, and genes of Notch, SHH, and WNT pathways are expressed in TICs of a variety of cancers [19], [20], [21]. VEGF is one of the putative targets of Hypoxia inducible factors-1α (HIF-1α) [22]. HIF1-α has also been shown to induce epithelial-mesenchymal transition (EMT) [23], which has recently been shown to be able to generate cells with stem cell properties in breast cancer [24]. Interestingly, we also found that CD133+ cells decreased in E-cadherin and increased in EMT markers such as N-cadherin, vimentin and fibronectin by quantitative RT-PCR and western blot analysis (Figure 2E). Importantly, the increase of EMT markers in CD133+ cells was also associated with an increase in the capacity for cell migration and invasion, characteristics of cells with increased EMT (Figure 2F). Thus, CD133+ cells possess EMT characteristics and increase embryonic stem cell markers and a HIF target gene.

### CD133+ cells are Resistant to Hypoxia and Serum Depletion Induced Apoptosis

**Figure 3:**
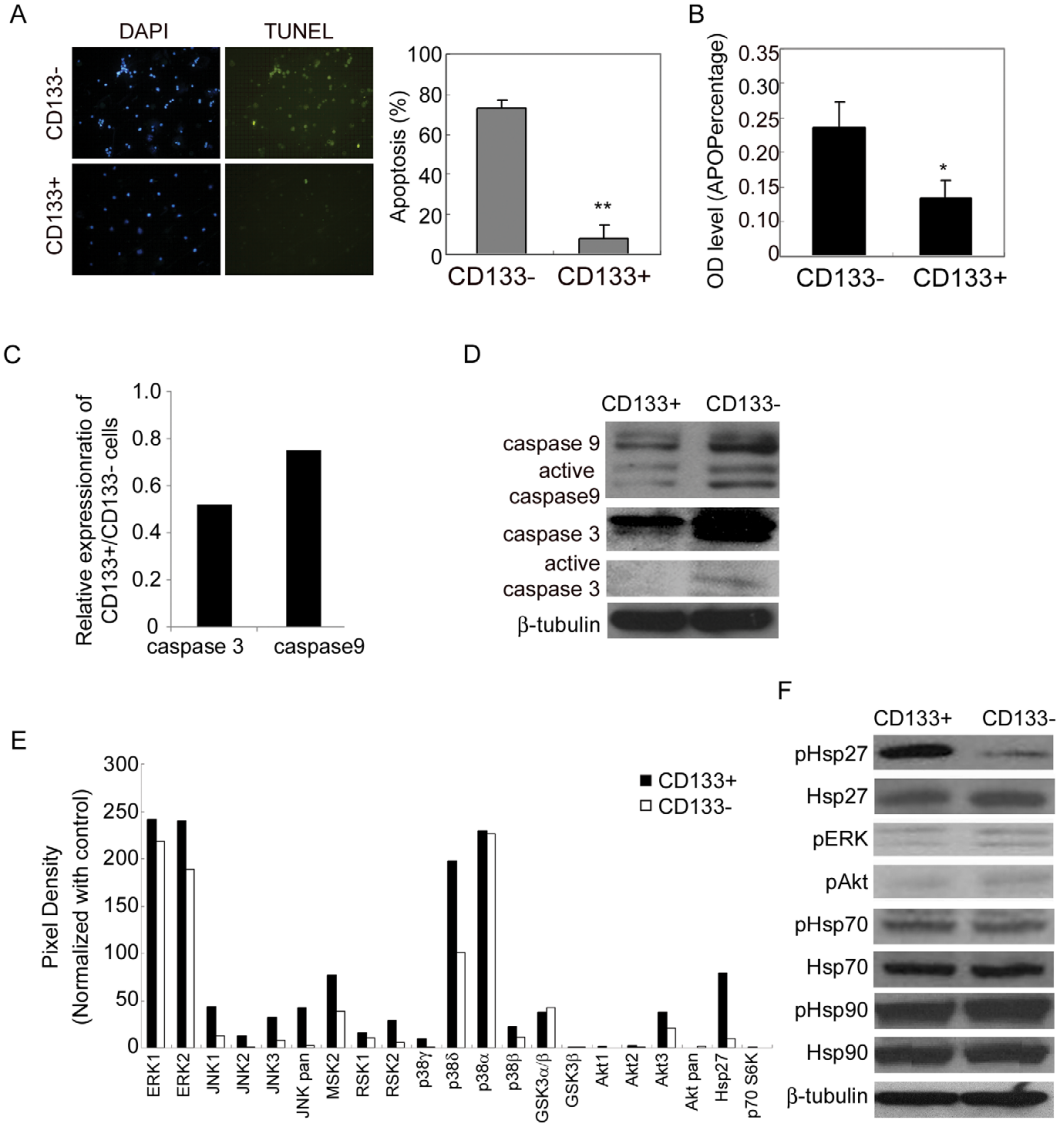
CD133+ cells resist hypoxia and serum depletion-induced apoptosis via activation of Hsp27. (A) TUNEL staining for apoptosis was performed after MACS separation of CD133− and CD133+ cells under hypoxia and serum depletion for 4 days. (B) CD133− and CD133+ cells under hypoxia and serum depletion for 3 days were harvested by MACS separation, reseeded under hypoxia and serum depletion, and APOPercentage assay was performed 16 h later. (C) Quantitative RT-PCR for mRNA levels. (D-F) Cell lysates of CD133+ and CD133− cells after MACS separation at day 4 of exposure to hypoxia and serum depletion were prepared and used for (D, F) immunoblot analysis for protein levels, and (E) MAPK phospho-antibody array for analyzing the levels of indicated signaling pathways. Graphs show each signaling after normalized with control. CD133+ cells decreased in the expression and the activation of caspase 9 and 3 and increased in the expression of phospho-Hsp27. Error bars represent standard deviations. (*p<0.05 and **p<0.01 as determined by the Student’s t test.) Data of HT-29 cells are shown as representative results.

We then investigated whether CD133+ cells might be resistant to hypoxia and serum depletion induced apoptosis. Indeed, CD133+ cells were found to be resistant to apoptosis as measured by the TUNEL assay, while CD133− cells were positive for TUNEL staining (Figure 3A). This notion was further supported when HT-29 cells were harvested after three days of hypoxia and serum depletion, and then the CD133+ and CD133− cell populations were respectively reseeded for more 16 h under the same conditions and analyzed using the APOPercentage assay. Here we found that in the CD133− population there was significantly more apoptosis than in the CD133+ cells (Figure 3B). Taken together, these results indicate that that CD133+ cells are resistant to hypoxia and serum depletion induced cell death. To elucidate the mechanism of CD133+ cells resistance to apoptosis, we first asked if this resistance occurred through a caspases-dependent apoptotic pathway. We found that expression of caspase genes such as caspase 9 and 3 was reduced in CD133+ cells compared with CD133− cells in both mRNA and protein levels, using quantitative RT-PCR (Figure 3C) and western blot analysis (Figure 3D), respectively. Furthermore, CD133+ cells also showed a reduced amount of the active cleaved forms of caspase 9 and caspase 3 (Figure 3D). However, we did not find any apparent difference between CD133+ and CD133− cells in the protein levels of phosphor-IKKα, nuclear NF-κB and its downstream targets, such as BCl-2 and survivin proteins (Figure S3). These results suggest that survival of CD133+ cells from hypoxia and serum depletion is mainly mediated by the lack of lack of caspase 9 and caspase 3 activation, but independent of NF- κB, BCL-2 and survivin.

### Hsp27 is Required for the Survival of TICs under Hypoxia and Serum Depletion

Since we found that there was less of the active form of caspase 9 and 3 (Figure 3D), we next investigated the molecular mechanisms responsible for the lack of activation of caspase 9 and 3 in CD133+ cells. To this end, we used rapid and sensitive Phospho-RTK (phospho-tyr) and Phospho-MAPK Arrays (phospho-ser/thr), and compared the phosphorylation of proteins in several different pathways in CD133+ cells to CD133− cells, under the conditions of hypoxia and serum depletion. In general there was no significant difference in tyrosine phosphorylation between the CD133+ and CD133− cells (Figure S4). On the other hand, there were several ser/thr kinase phosphorylations that were increased in CD133+ cells, the most obvious being Hsp27 (8.6 fold), when compared to CD133− cells (Figure 3E). Therefore, we further evaluated the involvement of Hsp27 in CD133+ cells resistance to apoptosis. The reliability of Phospho-MAPK Arrays was confirmed by western blot analysis and CD133+ indeed increased in Hsp27 activation, while the phosphorylation levels of ERK and Akt were not changed (Figure 3F). Therefore, these data suggest the resistance of CD133+ cells to apoptosis is associated with the activation of Hsp27.

### Hsp27 is Required for the Survival of TICs under Hypoxia and Serum Depletion

**Figure 4:**
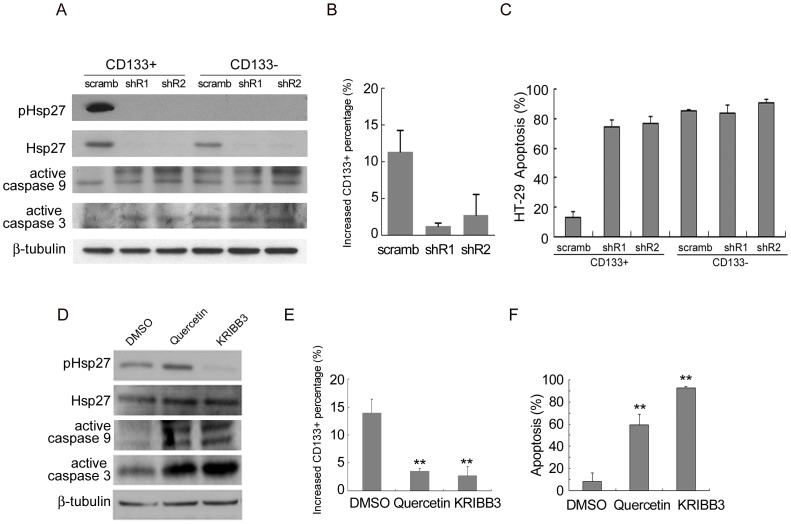
The involvement of Hsp27 activation in the anti-apoptosis pathway of CD133+ cells. Cells were lentivirally transfected with Hsp27 shRNA (shR1 and shR2) or scrambled shRNA, then exposed to hypoxia and serum depletion. CD133+ and CD133− cells were isolated using MACS separation 4 days later. (A) Immunoblot analysis for HT-29 protein levels of CD133+ and CD133− cells after MACS separation. (B) The percentage of CD133+ cells were analyzed by flow cytometry and the increased CD133+ percentage compared to CD133+ percentage in normoxia and growth medium was calculated. (C) Cells were re-exposed to hypoxia and serum depletion for 1 day, followed by TUNEL staining. (D-F) HT-29 cells were exposed to serum depletion and hypoxia in the presence of Quercetin, KRIBB3, or DMSO (vehicle control) for 4 days. (D) CD133+ cell were isolated and the protein levels were analyzed by western blotting. (E) The increased percentage of CD133+ cells and (F) apoptosis of CD133+ cells were analyzed by flow cytometry and TUNEL staining, respectively. Error bars represent standard deviations. (**p<0.01 compared with the scrambled as determined by the Student’s t test.)

To investigate whether Hsp27 activity is indeed important to protect CD133+ population from apoptosis, we first used two Hsp27-shRNAs and showed that repression of Hsp27 (Figure 4A) indeed sensitized apoptosis in the CD133+ population in responding to hypoxia and serum depletion. Furthermore, Hsp27-specific shRNA induced a decrease in the increased CD133+ percentage under hypoxia and serum free medium conditions, as compared with the scrambled shRNA (Figure 4B). An increase in apoptosis was also observed with two Hsp27-specific shRNAs in CD133+ cells of HT-29 (Figure 4C). Similar results were also observed in the primary culture CCS (Figure S5A). Western blot analysis also revealed an increase in the cleavage of caspase 9 and 3 by Hsp27-specific shRNAs (Figure 4A). In addition, two structurally distinct Hsp27 inhibitors, Quercetin and KRIBB3 (specific for Hsp27 phosphorylation) [25], also increased the cleavage of caspases 9 and 3 in HT-29 cells (Figure 4D) and CCS cells (Figure S5B) and induced a decrease in the increased percentage of CD133+ cells under hypoxia and serum depletion (Figure 4E) and resulted in apoptosis in CD133+ cells (Figure 4F), As expected, the PI3K-Akt inhibitor, LY294002 and ERK inhibitor, U0126 did not induce these effects (Figure S5C and S5D). It should be mentioned that Quercetin is known to inhibit Hsp27, Hsp70 and Hsp90. Thus, we also examined the activation of Hsp90 and Hsp70 in the CD133+ and CD133− populations (Figure 3F). The results demonstrate that there is no difference in the pHsp70 and pHsp90 between the two populations of cells. Thus, among the three Hsp proteins, Hsp27 seems to be the only that is activated in CD133+ cells, thus the Quercetin effect is likely caused by the inhibition of Hsp27. Taken together, these results suggest that Hsp27 is required for the survival of TICs under hypoxia and serum depletion.

### Decrease in PP2A Activity Causes Activation of Hsp27 in CD133+ cells

**Figure 5:**
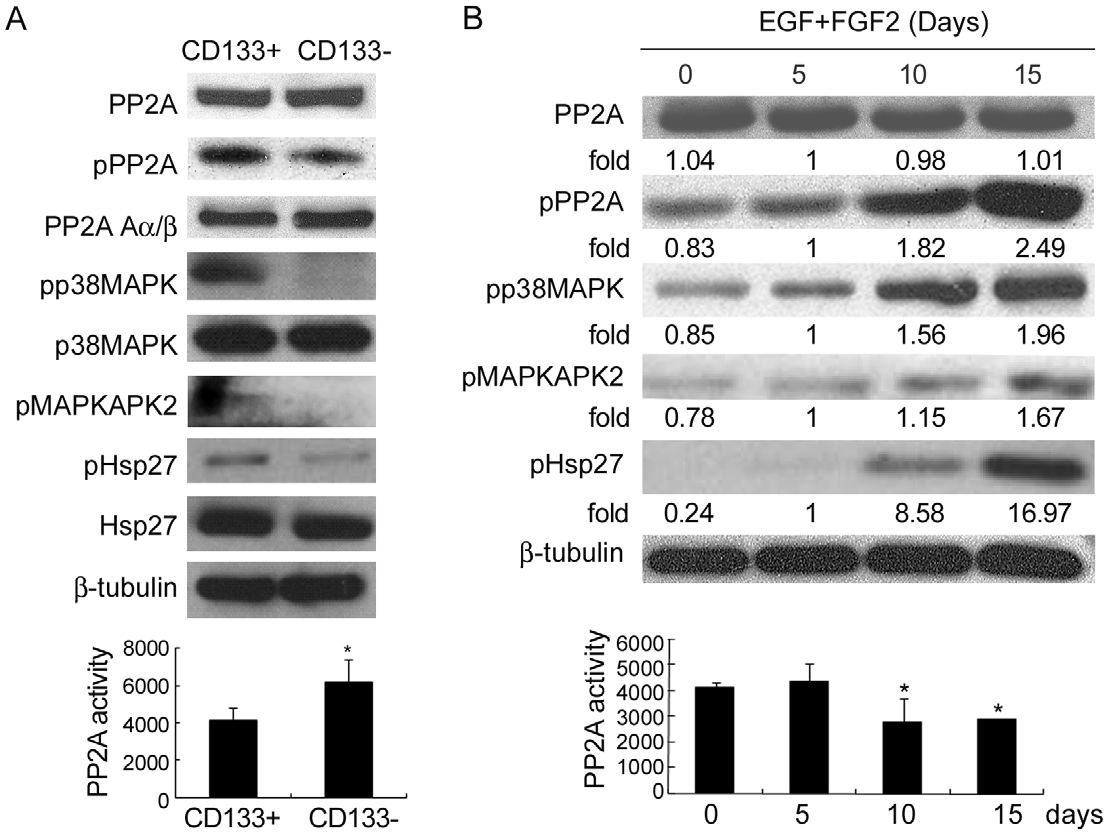
Decrease in PP2A activity increases phosphorylation of p38MAPK, pMAPKAPK2 and Hsp27 in CD133+ cells. (A upper panel) Immunoblot analysis and (A lower panel) PP2A activity analysis for HT-29 CD133+ and CD133− cells after MACS separation at day 4 of exposure to hypoxia and serum depletion. (B upper panel) Immunoblot analysis and (B lower panel) PP2A activity of CCS cells cultured under serum depletion in the presence of EGF (10 ng/mL) and FGF2 (10 ng/mL) (EGF+FGF2) for indicated time periods. The fold values are relative to the band intensity of the 5 day treatment.

Upon stimulation by stress, p38MAPK is phosphorylated, which then phosphorylates MAPKAPK2 to activate Hsp27 [26]. Therefore, in order to elucidate the upstream signalling of Hsp27 activation in CD133+ cells, we examined the involvement of p38MAPK and MAPKAPK2. We found that activation of both p38MAPK and MAPKAPK2 was greater in CD133+ than CD133− cells (Figure 5A). Protein phosphatase 2A (PP2A), a general protein phosphatase, functions as a tumor suppressor [27], [28]. A decrease in PP2A activity was reported to promote anchorage-independent growth and tumor formation after *in vivo* transplantation, which are the two characteristics of cancer initiating cells [27]. Dephosphorylation of Hsp27 by PP2A was observed in human fibroblasts when treated with okadaic acid (OA), a specific PP2A inhibitor [29]. However, it is not yet clear how PP2A can dephosphorylate Hsp27. We therefore asked whether PP2A is involved in the activation of Hsp27 in CD133+ TICs through the p38MAPK-MAPKAPK2 pathway. To this end, we observed a decrease in PP2A activity and consistent with the decrease in activity there was an increase in phosphorylation of PP2A (Tyr307) in CD133+ cells (Figure 5A). The protein and mRNA levels of PP2A Aα/β and B subunits, PP1 and PP2B and the phosphorylation level of PP1 were not significantly different between CD133+ and CD133− cells (Figure 5A, Figure S6A left panel and Figure S6B). Need to note, the activation of the PP2A-p38-Hsp27 pathway by CD133+ cells rather than CD133− cells was not found before exposure to hypoxia and serum starvation (Figure S6C). Additionally, we found the same changes with PP2A activity and phosphorylation in CCS cells treated (reduced serum, +EGF, +FGF2) to enrich TICs for 10 and 15 days, which similarly caused the activation of p38MAPK, MAPKAPK2 and Hsp27 (Figure 5B). Separation of CD133+ and CD133− cells via sorting, followed by western blot analysis further revealed the increase in the phosphorylation levels of PP2A, p38MAPK, MAPKAPK2 and Hsp27 in spheroid culture was observed in CD133+ cells rather in CD133− cells (Figure S6D and S6E). The protein level of PP1 and PP2B were not significantly different at 5, 10, and 15 days of enrichment of TICs (Figure S6A right panel). Similar results were observed in HT-29 and HCW cells (data not shown). Inhibition of PP2A activity by incubation with the PP2A inhibitor, Calyculin A (CA) or OA also activated p38MAPK, MAPKAPK2 and Hsp27 in the parental colorectal cells (Figure S6F and S6G). Together, these results suggest that the activation of p38MAPK-MAPKAPK2-Hsp27 pathway in TICs may be due to their decrease in PP2A activity. To further support the notion that Hsp27 activation by a decrease in PP2A activity in TICs is mediated by the p38MAPK-MAPKAPK2 pathway, cells were treated without or with CA or OA in the absence or presence of p38MAPK-specific, SB203580. Treatment of CA or OA induced the activation of p38MAPK-MAPKAPK2-Hsp27 pathway, whereas the activation of MAPKAPK2 and Hsp27 by CA or OA was completely blocked by the p38 MAPK inhibitor, SB203580 (Figure S6H and S6I), suggesting that activation of Hsp27 through suppression of PP2A requires p38MAPK activity. To investigate the possible mechanism of PP2A phosphorylation, we examined the involvement of c-Src, which phosphorylates the C subunit of PP2A (Tyr-307), and Jak2, possible upstream signals of PP2A [30], [31]. We found that the phosphorylation levels of c-Src (Tyr416) and Jak2 were increased at 10 days of enrichment of TICs (Figure S7A). PP2, a selective inhibitor of c-Src, but not PP3 (inactive analog) or the inhibitor of Jak2, AG490, inhibited the phosphorylation of PP2A, p38MAPK and Hsp27 (Figure S7B), suggesting the activation of c-Src was involved in PP2A phosphorylation. In addition, PP2 but not PP3 inhibited the formation of sphere in spheroid culture (Figure S7C), suggesting the involvement of c-Src in activation of the antiapoptosis pathways in TICs. Taken together, these results in TICs demonstrate that activation of the p38MAPK-MAPKAPK2-Hsp27 pathway, associated with decreased PP2A activity and increased c-Src activation, inhibits the cleavage of caspase 9 and 3 and enhances the survival of TICs under hypoxia and serum depletion conditions.

### Decrease in PP2A and Increase in Hsp27 Activation in Other Solid Tumors

**Figure 6:**
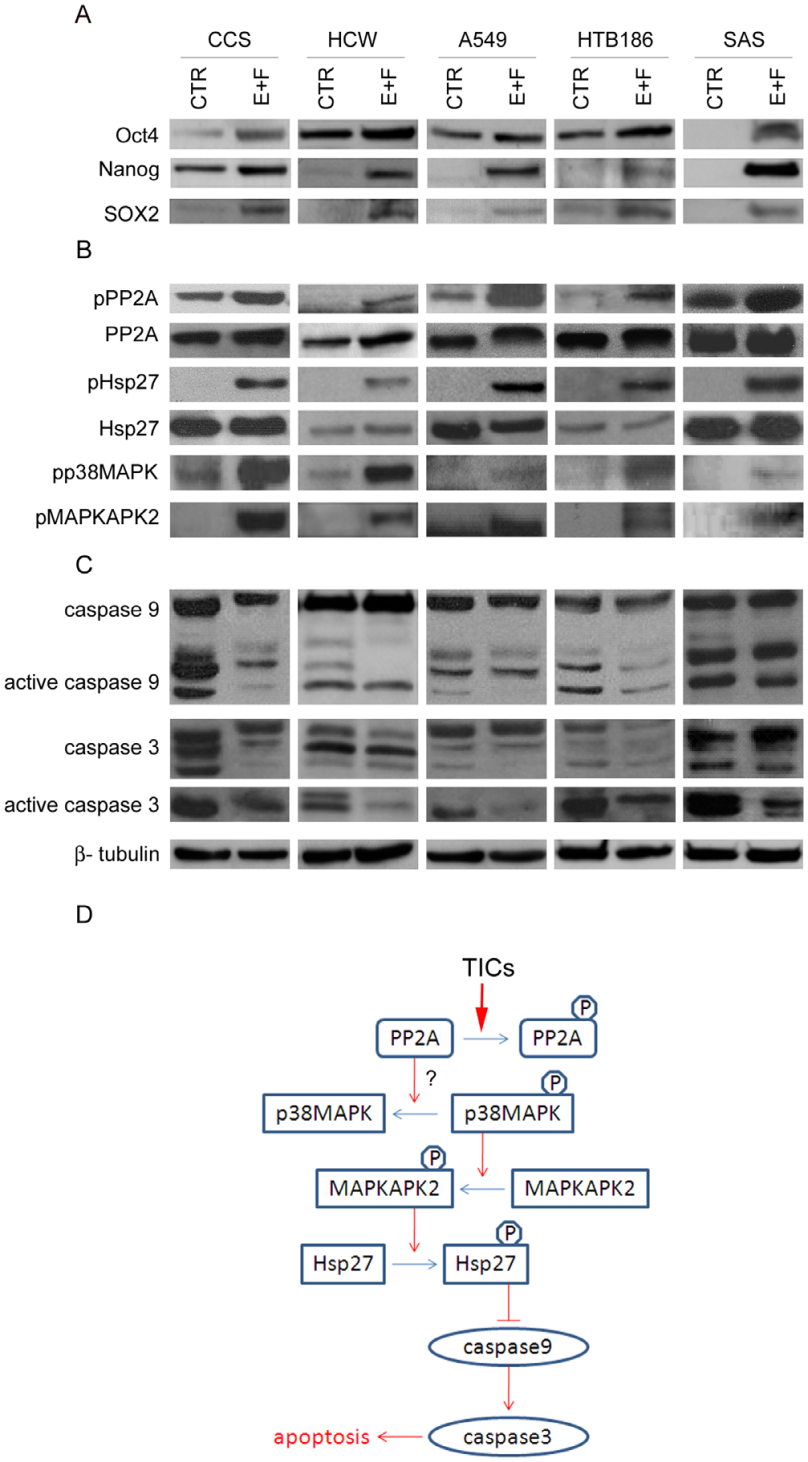
Enrichment of TICs decreases PP2A activity and increases Hsp27 activation in other solid tumors. CCS and HCW colorectal cancer cells, A549 lung cancer cells, HTB186 medulloblastoma cells and SAS, oral cancer cells were continually cultured under serum depletion in the presence of EGF (10 ng/mL) and FGF2 (10 ng/mL) to enrich TICs (E+F) or in serum-containing medium (CTR) as a control. (A) Immunoblots for pluripotent markers. (B) Immunoblots for phosphorylated PP2A (pPP2A), PP2A and Hsp27. (C) Immunoblots for caspase 9 and 3. (D) Schema demonstrates the antiapoptosis pathway of colorectal TICs in response to in vitro hypoxia and serum depletion.

Since the Hsp27 pathway was constitutively activated in TICs of colorectal cancer, we next asked whether similar pathways were activated in TICs of other tumors. We examined the activation of Hsp27 in TICs of solid tumors such as lung cancer, brain cancer and oral cancer. Similar to CCS colorectal cancer cells, after enrichment of TICs with the increased expression of markers such as Oct4, Nanog and SOX2 (Figure 6A), there was an increase in PP2A phosphorylation, and an increase in p38MAPK, MAPKAPK2 and Hsp27 activation in HCW, a primary liver metastasis of colorectal cancer [15], A549, a lung adenocarcinoma cell line, HTB-186, medulloblastoma cells and SAS, primary oral cancer cells (Figure 6B). Consistently, the expression and cleavage of caspase 3 and 9 was also decreased in the TICs of these cell lines (Figure 6C). Thus, these results suggest that the proposed activation of p38MAPK-MAPKAPK2-Hsp27 pathway through the decrease in PP2A expression may be common in the *in vitro* enriched TICs of many different cancer cell types.

## Discussion

In this current study we analyzed the response of colon cancer cells to hypoxia and serum depletion, the consequential conditions occurred after antiangiogenesis, irradiation and chemotherapy [11], [12], and discovered the CD133+ TICs were resistant to hypoxia and serum depletion. It should also be mentioned that the percentage of CD44+ and CD166+ cells, which are known to be colon cancer initiating cells and are resistant to chemotherapy [16], was not increased under hypoxia and serum free conditions, raising the issue of whether there are two different populations of TICs, i.e. CD133+ and CD44+/CD166+. Previously, TrkA-Trk-A, HGFR, Erb-B4 have been shown increased in TICs in a variety of tumors, however, these protein levels were not different between non-TICs and TICs enriched by hypoxia and serum depletion, suggesting there are more than one subpopulations of TICs in tumor, each can be enriched by different conditions. It will be interesting to investigate further the interrelationship between CD133+ and CD44+/CD166+ and their relevance as TIC markers. Needless to say, the current study identifies CD133+ cells as a target for colon cancer therapy.

Hsp27 has previously been reported to induce apoptosis resistance in rat colon cancer cell line [32] and to enhance antiapoptotic protein expression in murine normal and transformed cells [33]. It is known that Hsp27 can inhibit apoptosis by inhibiting caspase 9, through the interaction with cytochrome c and prevention of the formation of the apoptosome complex, and caspase 3, through interacting with pro-caspase 3 molecules [32], [34]. Here, we found that activation of Hsp27 inhibited cleavage of caspase 9 and 3 in the TICs of colorectal cancer. Upon stimulation by stress, p38MAPK is phosphorylated, which then phosphorylates MAPKAPK2 to phosphorylate and activate Hsp27 [26]. It is also known that phosphorylation of Hsp27 is induced through the inhibition of PP2A [29], however, the details of the Hsp27 and PP2A relationship have not been characterized. Our current study provides a likely linkage between PP2A and Hsp27 through the p38MAPK-MAPKAPK2-Hsp27 pathway (Figure 6D).

Targeting Hsp27 in TICs may help to develop new strategies for reducing tumor resistance to modern cancer therapy such as antiangiogenesis, irradiation and chemotherapy, but, until now, there are only two drugs, Quercetin and KRIBB3, reported to inhibit Hsp27 activity [35]. In the current study we use both Quercetin and KRIBB3 to inhibit Hsp27. In addition, we used shRNA and obtained results consistent with two the inhibitors, supporting the notion that CD133+ tumor cells preferentially activate the p38MAPK-MAPKAPK2-Hsp27 pathway probably through suppression of PP2A to inhibit caspase 9 and 3 cleavage.

PP2A is a ubiquitously expressed protein serine/threonine phosphatase that accounts for a large fraction of the phosphatase activity in eukaryotic cells [27]. Recent evidence indicates that suppression of PP2A protein or activity is needed to push immortalized cells to complete transformation [36], [37]. Here we show that PP2A activity is suppressed in TICs in a variety of cancer cell types. Suppression of PP2A also resulted in anchorage-independent cell growth and tumor formation in mice, two essential factors for tumor initiation. Therefore, suppression of PP2A in TICs may enable the tumor initiation capacity in TICs. Similar with a recent report [38], we also identified the activation of c-Src in TICs and further demonstrated that treatment of TICs with specific inhibitor to inactivate c-Src activity induced an increase in PP2A activity, thereby contributing to the inactivation of the p38MAPK-MAPKAPK2-Hsp27 pathway and inhibition of the formation of sphere in spheroid culture.

Since hypoxia and serum or nutrition deprivation occur *in vivo* following tumor enlargement with central necrosis and a lot of cancer treatments such as antiangiogenesis, irradiation and chemotherapy, thus the current data may be applied for the development of new strategies in treating cancer. To apply the current findings for future targeting of TICs, we need to perform *in-vivo* tumor analysis and show the same signaling pathway is also activated in TICs upon exposure to *in vivo* conditions associated with hypoxia and serum or nutrition deprivation. In the several cancer types investigated here, increase in the activation of p38MAPK-MAPKAPK2-Hsp27 pathway was observed in their TICs. As a result, these Hsp27-activated TICs may contribute to their survival and recurrence from the current cancer therapies. Thus, activation of PP2A or inactivation of the p38MAPK-MAPKAPK2-Hsp27 pathway may have potential use for cancer therapy by suppression of their TIC population.

## Supporting Information

Figure S1 **Bulk and CD133+ cells form colorectal tumor when injected subcutaneously into the flanks of NOD/SCID mice.** (A) The rate of tumor formation by injection of indicated numbers of HT-29 bulk tumor cells, or CD133+ cells and CD133− cells isolated from 4-day culture under hypoxia and serum depletion conditions. (B) Tumors were removed for H&E staining and immunohistochemical staining for CD133, CK20, CDX2, CK7 and β-catenin. Scale bar, 50 µm. (106 bulk cells and 103 CD133+ cells were injected in this case, 104–105 CD133− cells injected did not form tumor.) (C) CD133+ cells and CD133− cells isolated from xenograft tumor formed by CD133+ cells were cultured under normoxia and growth medium for 4 days, followed by assay of the percentage of CD133+ and CD133− cells using flow cytometric analysis.
(TIF)

Figure S2 **Comparisons in gene expression between CD133+ and CD133**− **cells.** CD133+ and CD133− cells were isolated from 4-day culture of HT-29 cells under hypoxia and serum depletion conditions. Quantitative RT-PCR was assayed for the expression of indicated genes.
(TIF)

Figure S3 **Comparisons of proteins associated apoptosis between CD133+ and CD133**− **cells.** Cell lysates of CD133+ and CD133− cells after MACS separation of HT-29 cells at day 4 of exposure to hypoxia and serum depletion were prepared and used for (A) immunoblot analysis for protein levels and (B left panel) immuonfluorescence studies and (B right panel) quantitative data. Scale bar, 100 µm.
(TIF)

Figure S4 **Tyrosine Kinase Phospho-Antibody array analysis.** CD133+ and CD133− cells isolated from HT-29 cells after 4 days under hypoxia and serum depletion. Tyrosine kinase Phospho-Antibody array was used to detect phosphorylation of receptor tyrosine kinases and signaling molecules. Dot blots for one experiment with duplicate for each signaling are shown. Bar graph represents the average of the two pixel density from each array. Representative histograms of two dot blots for each signaling are shown in the lower panel.
(TIF)

Figure S5 **The involvement of Hsp27 activation in the anti-apoptosis pathway of CD133+ cells.** CCS cells were lentivirally transfected with Hsp27 shRNA (shR1 and shR2) or scrambled shRNA, then exposed to hypoxia and serum depletion. CD133+ and CD133− cells were isolated using MACS separation 4 days later. (A) Cells were re-exposed to hypoxia and serum depletion for 1 day, followed by TUNEL staining. (B) CCS cells were exposed to serum depletion (SF) and hypoxia (Hyp) in the presence of Quercetin, KRIBB3, or DMSO (vehicle control), and 4 days later, the CD133+ cells were isolated for immunoblotting. (C, D) HT-29 cells were exposed to SF and Hyp conditions for 4 days in the presence of indicated inhibitors. The incrased percentage of (C) CD133+ cells and (D) apoptosis of CD133+ cells were analyzed by flow cytometry and TUNEL staining, respectively. Error bars represent standard deviations. (**p<0.01 compared with the scrambled as determined by the Student’s t test.)
(TIF)

Figure S6 **Decrease in PP2A increases phosphorylation of p38MAPK, pMAPKAPK2 and Hsp27 in enriched tumor initiating cells.** (A left panel) Immunoblot analysis for HT-29 CD133+ and CD133− cells after MACS separation at day 4 of exposure to hypoxia and serum depletion. (A right panel) Immunoblots of CCS cells cultured under serum depletion in the presence of EGF (10 ng/mL) and FGF2 (10 ng/mL) (EGF+FGF2) for indicated time periods. (B) Quantitative RT-PCR for mRNA levels of HT-29 CD133+ and CD133− cells after MACS separation at day 4 of exposure to hypoxia and serum depletion. Bars indicate the expression ratio. (C) Immunoblots of HT29 CD133+ and CD133− cells under normal growth condition (FBS/Nor). (D, E) Flow cytometry and sorting for CD133+ and CD133− fractions in condition of EGF+FGF2. Dotted line represents isotype control. Ranges for sorting are shown in the upper region of the histogram. (E) Cell lysates were subjected for western blotting analysis. (F, G) HT-29 or CCS cells culture in the presence of Calyculin A (CA) and Okadaic acid (OA) at indicated concentrations or as a control, with DMSO, and immunoblot analysis was done at 30 min later. (H, I) HT-29 and CCS cells cultured in the presence of CA or OA with or without SB203580 at 50 µM (SB) or as a control, with DMSO, and immunoblot analysis was done at 30 min later.
(TIF)

Figure S7 **The involvement of c-Src activation in reducing PP2A activity and inducing sphere formation.** (A) Immunoblots of CCS cells cultured in control growth medium or under serum depletion in the presence of EGF (10 ng/mL) and FGF2 (10 ng/mL) (EGF+FGF2) for 10 days. (B) Immunoblots of CCS cell grown under serum depletion in the presence of EGF (10 ng/mL) and FGF2 (10 ng/mL) (EGF+FGF2) for 15 days with DMSO (vehicle control), PP2 (10 µM), PP3 (10 µM) or AG490 (10 µM). Fold of pPP2A was normalized with the level of β-tubulin. (C) Spheres formed by CCS cell grown under serum depletion in the presence of EGF (10 ng/mL) and FGF2 (10 ng/mL) (EGF+FGF2) for 15 days with DMSO (vehicle control), PP2 (10 µM), and PP3 (10 µM) (n = 3). (*p<0.05 and **p<0.01 compared with DMSO as determined by the Student’s t test.).
(TIF)
