# Supplementary figures and images for: Survival of Cancer Stem Cells under Hypoxia and Serum Depletion via Decrease in PP2A Activity and Activation of p38-MAPKAPK2-Hsp27
Source: PLoS One. 2012 Nov 20;7(11):e49605. doi: 10.1371/journal.pone.0049605 (PMC3502468; doi:10.1371/journal.pone.0049605)

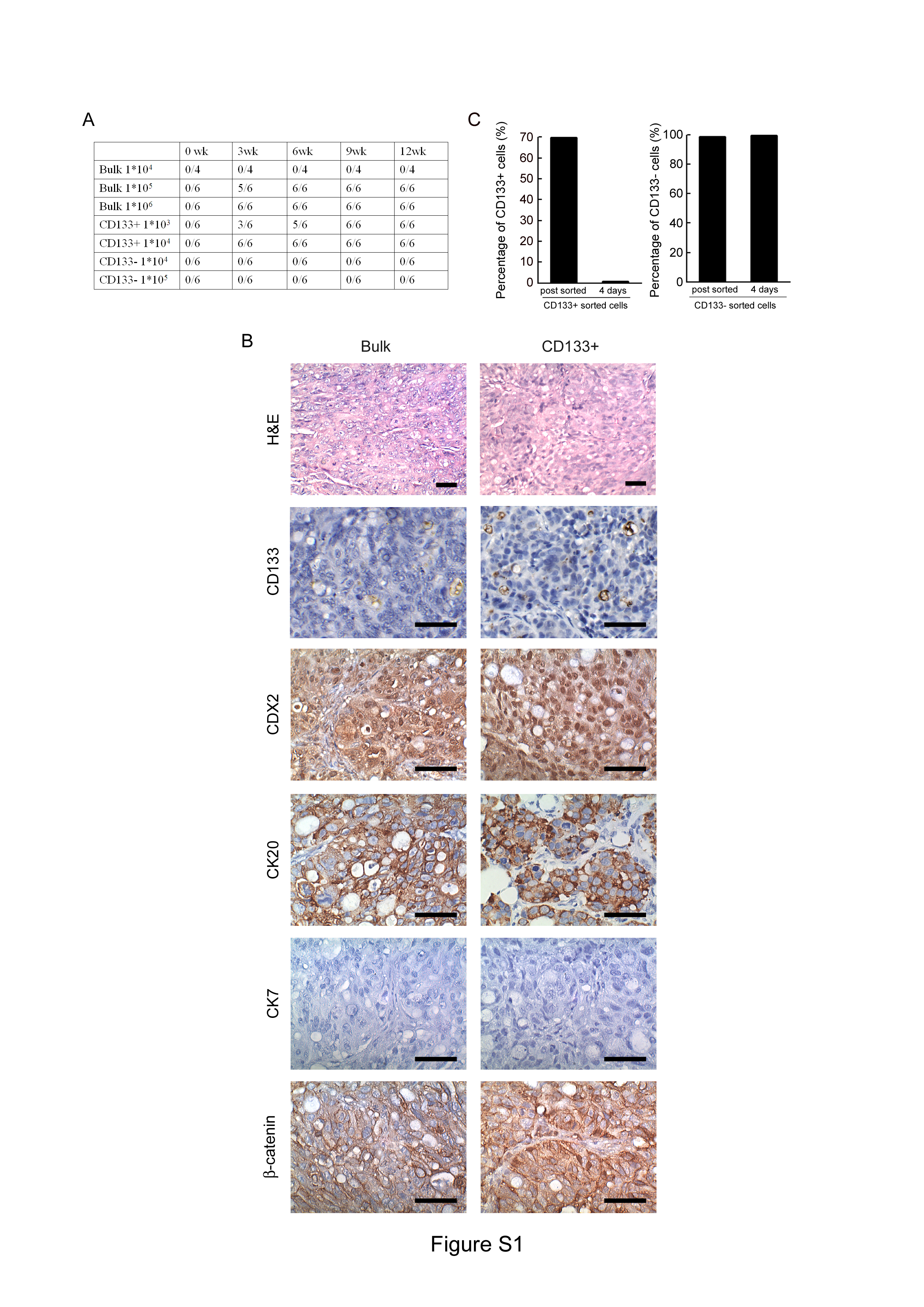

Supplement: Figure S1 — Bulk and CD133+ cells form colorectal tumor when injected subcutaneously into the flanks of NOD/SCID mice. (A) The rate of tumor formation by injection of indicated numbers of HT-29 bulk tumor cells, or CD133+ cells and CD133− cells isolated from 4-day culture under hypoxia and serum depletion conditions. (B) Tumors were removed for H&E staining and immunohistochemical staining for CD133, CK20, CDX2, CK7 and β-catenin. Scale bar, 50 µm. (106 bulk cells and 103 CD133+ cells were injected in this case, 104–105 CD133− cells injected did not form tumor.) (C) CD133+ cells and CD133− cells isolated from xenograft tumor formed by CD133+ cells were cultured under normoxia and growth medium for 4 days, followed by assay of the percentage of CD133+ and CD133− cells using flow cytometric analysis. (TIF) [file pone.0049605.s001.tif]

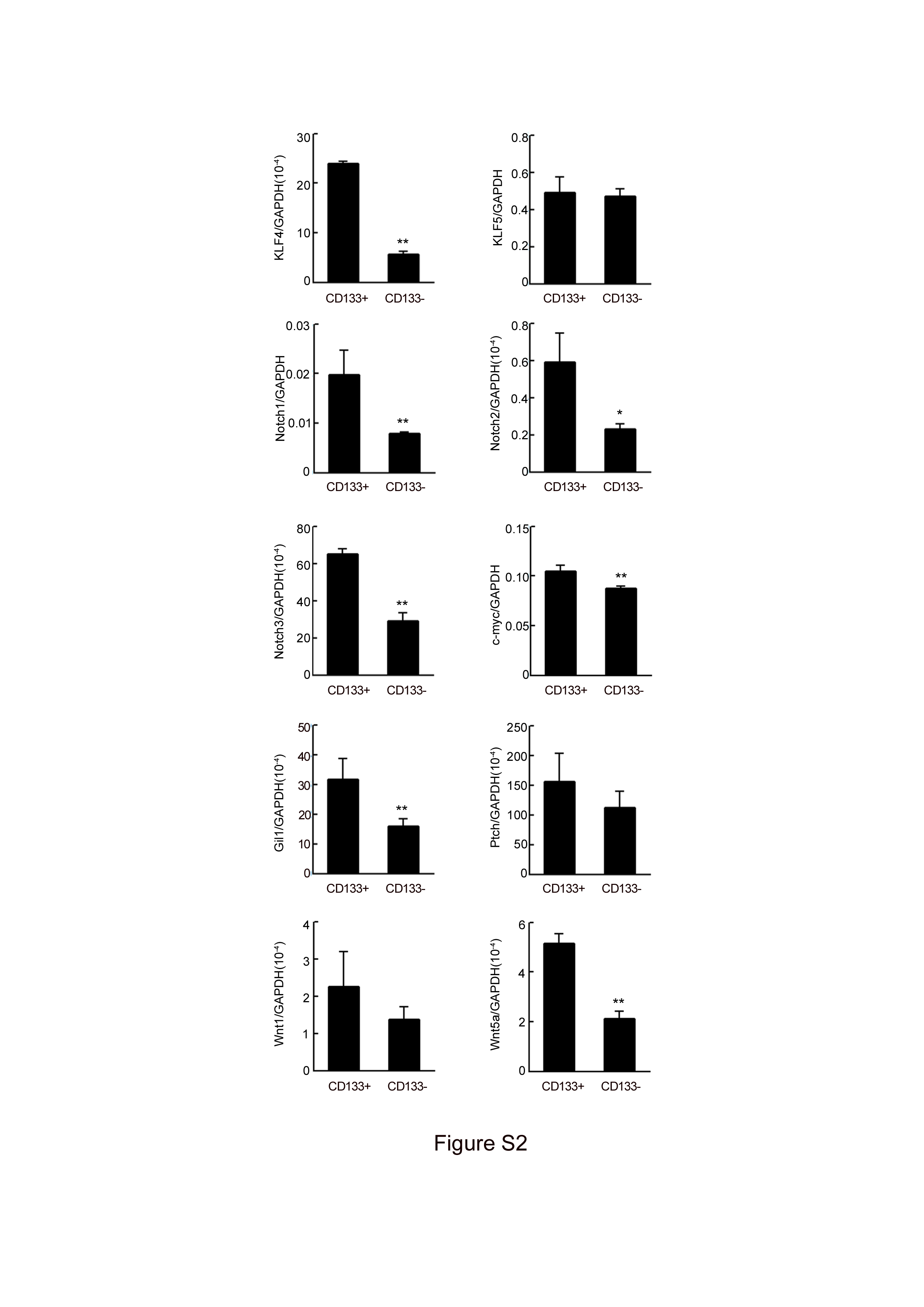

Supplement: Figure S2 — Comparisons in gene expression between CD133+ and CD133− cells. CD133+ and CD133− cells were isolated from 4-day culture of HT-29 cells under hypoxia and serum depletion conditions. Quantitative RT-PCR was assayed for the expression of indicated genes. (TIF) [file pone.0049605.s002.tif]

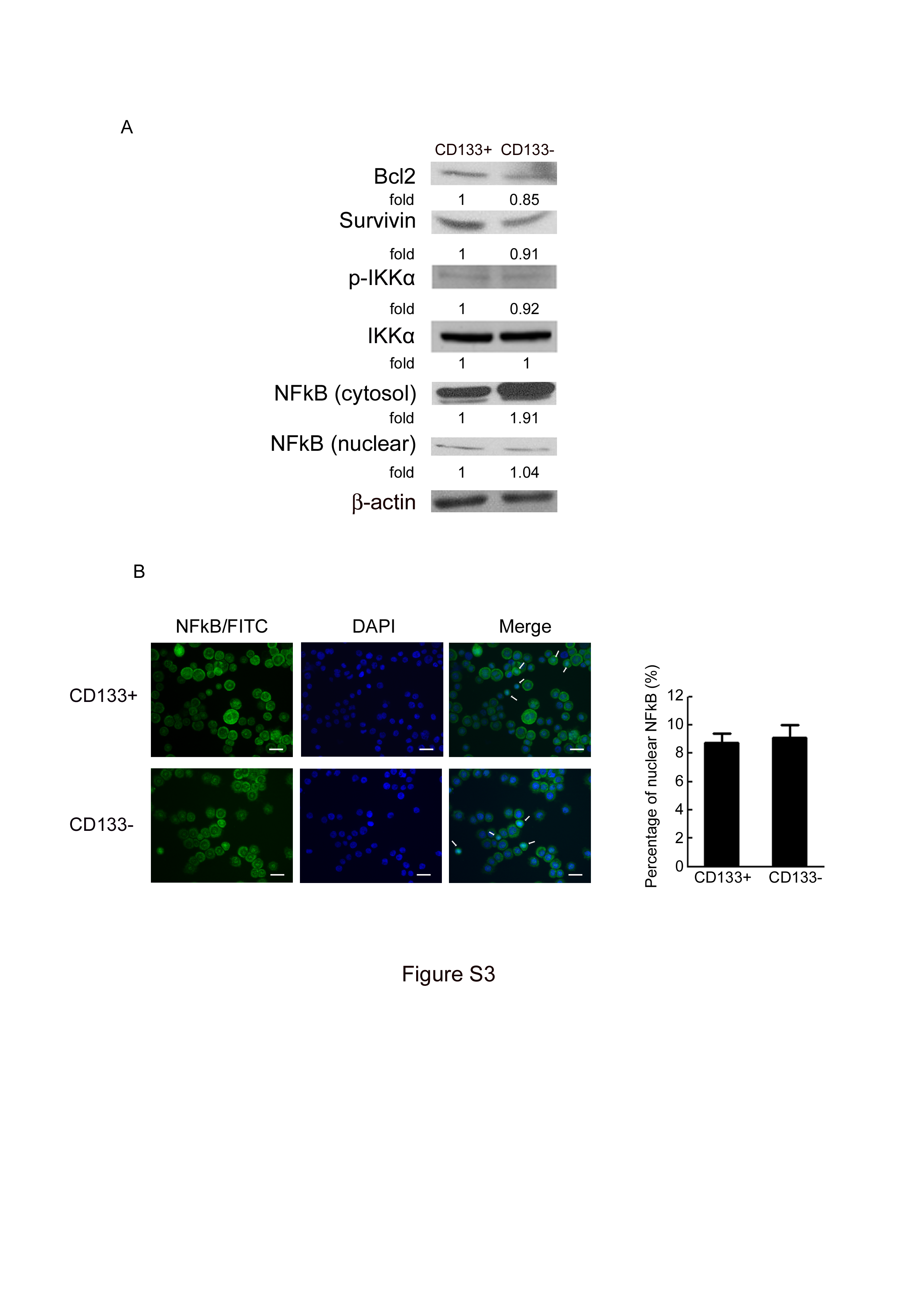

Supplement: Figure S3 — Comparisons of proteins associated apoptosis between CD133+ and CD133− cells. Cell lysates of CD133+ and CD133− cells after MACS separation of HT-29 cells at day 4 of exposure to hypoxia and serum depletion were prepared and used for (A) immunoblot analysis for protein levels and (B left panel) immuonfluorescence studies and (B right panel) quantitative data. Scale bar, 100 µm. (TIF) [file pone.0049605.s003.tif]

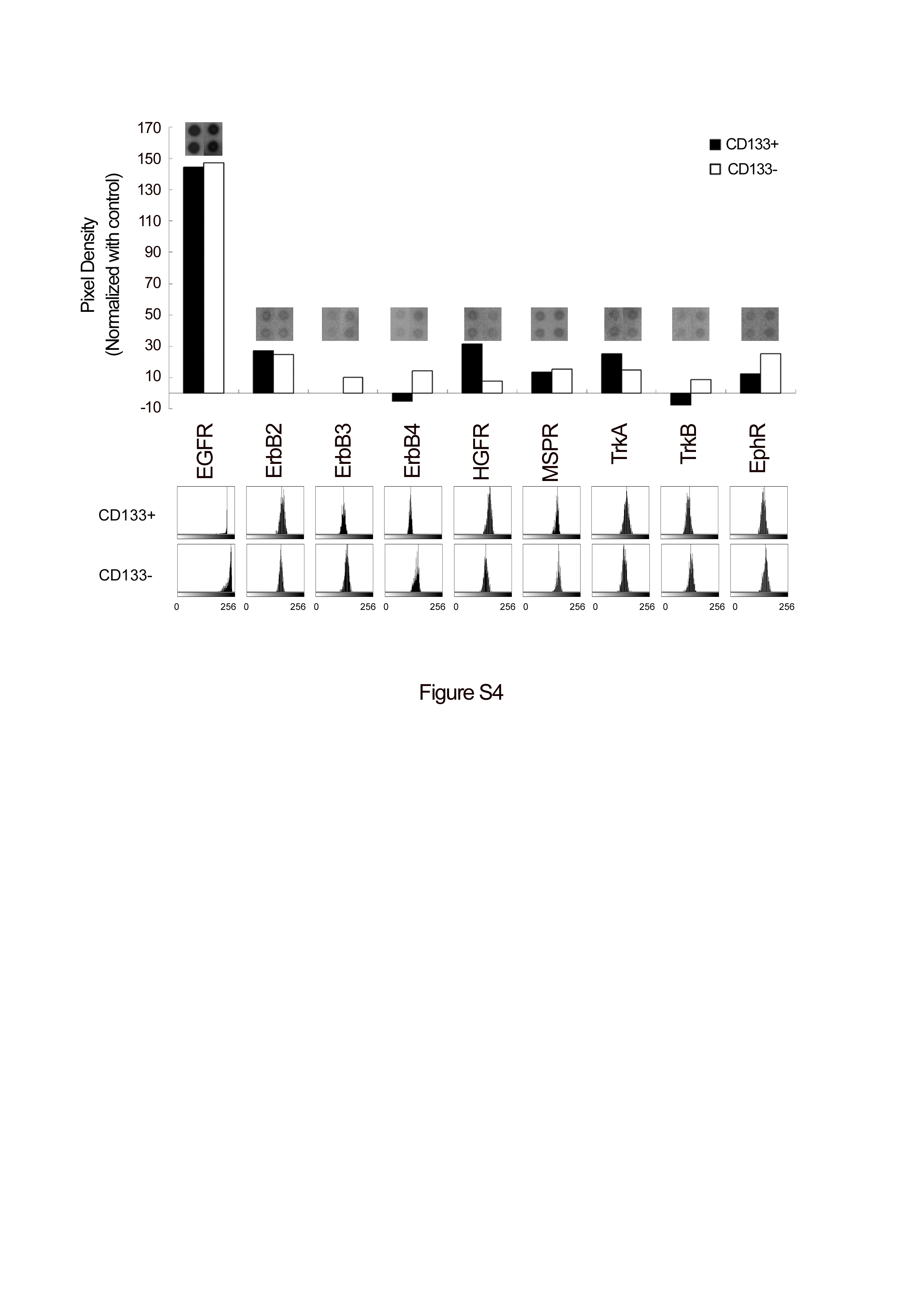

Supplement: Figure S4 — Tyrosine Kinase Phospho-Antibody array analysis. CD133+ and CD133− cells isolated from HT-29 cells after 4 days under hypoxia and serum depletion. Tyrosine kinase Phospho-Antibody array was used to detect phosphorylation of receptor tyrosine kinases and signaling molecules. Dot blots for one experiment with duplicate for each signaling are shown. Bar graph represents the average of the two pixel density from each array. Representative histograms of two dot blots for each signaling are shown in the lower panel. (TIF) [file pone.0049605.s004.tif]

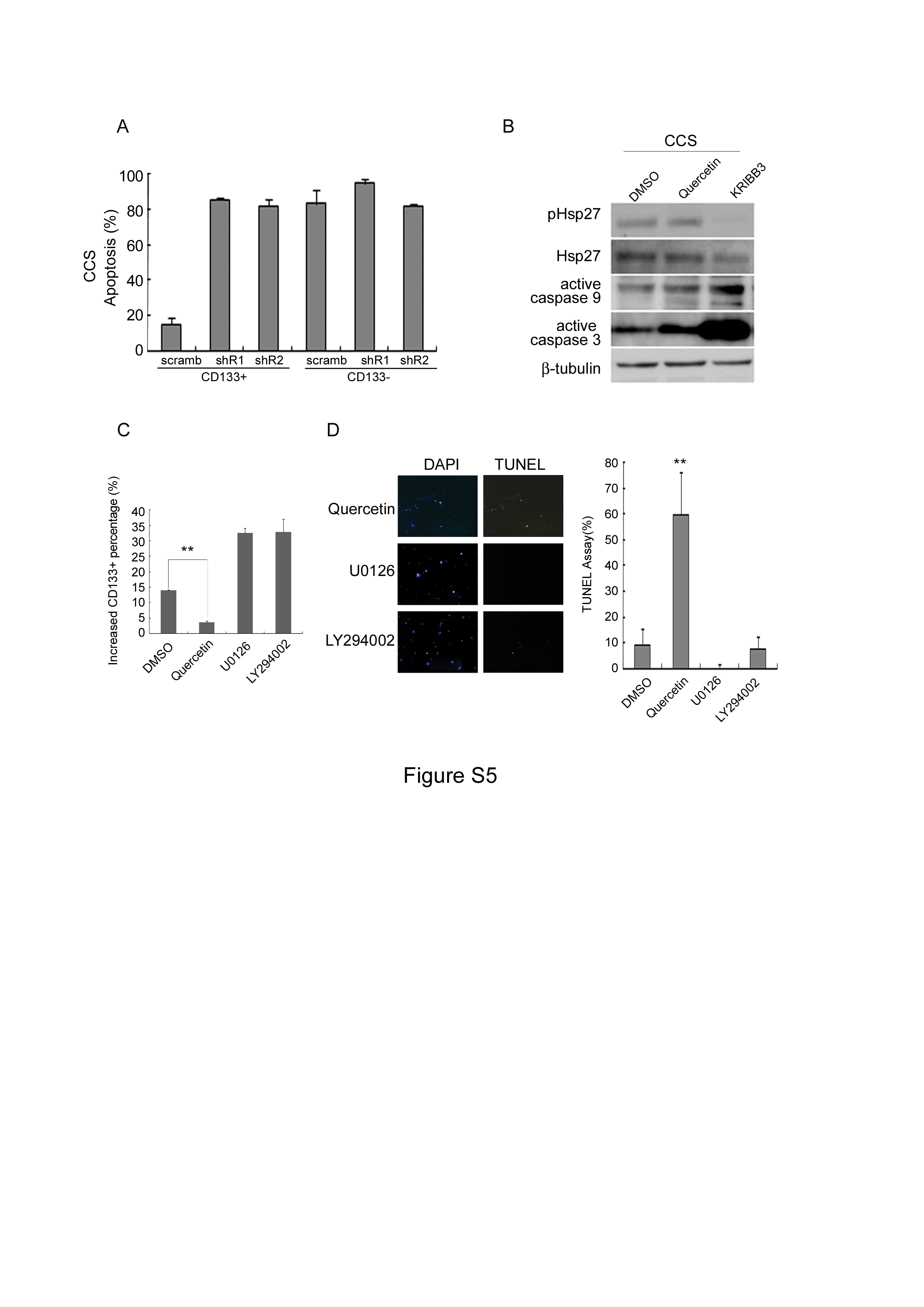

Supplement: Figure S5 — The involvement of Hsp27 activation in the anti-apoptosis pathway of CD133+ cells. CCS cells were lentivirally transfected with Hsp27 shRNA (shR1 and shR2) or scrambled shRNA, then exposed to hypoxia and serum depletion. CD133+ and CD133− cells were isolated using MACS separation 4 days later. (A) Cells were re-exposed to hypoxia and serum depletion for 1 day, followed by TUNEL staining. (B) CCS cells were exposed to serum depletion (SF) and hypoxia (Hyp) in the presence of Quercetin, KRIBB3, or DMSO (vehicle control), and 4 days later, the CD133+ cells were isolated for immunoblotting. (C, D) HT-29 cells were exposed to SF and Hyp conditions for 4 days in the presence of indicated inhibitors. The incrased percentage of (C) CD133+ cells and (D) apoptosis of CD133+ cells were analyzed by flow cytometry and TUNEL staining, respectively. Error bars represent standard deviations. (**p<0.01 compared with the scrambled as determined by the Student’s t test.) (TIF) [file pone.0049605.s005.tif]

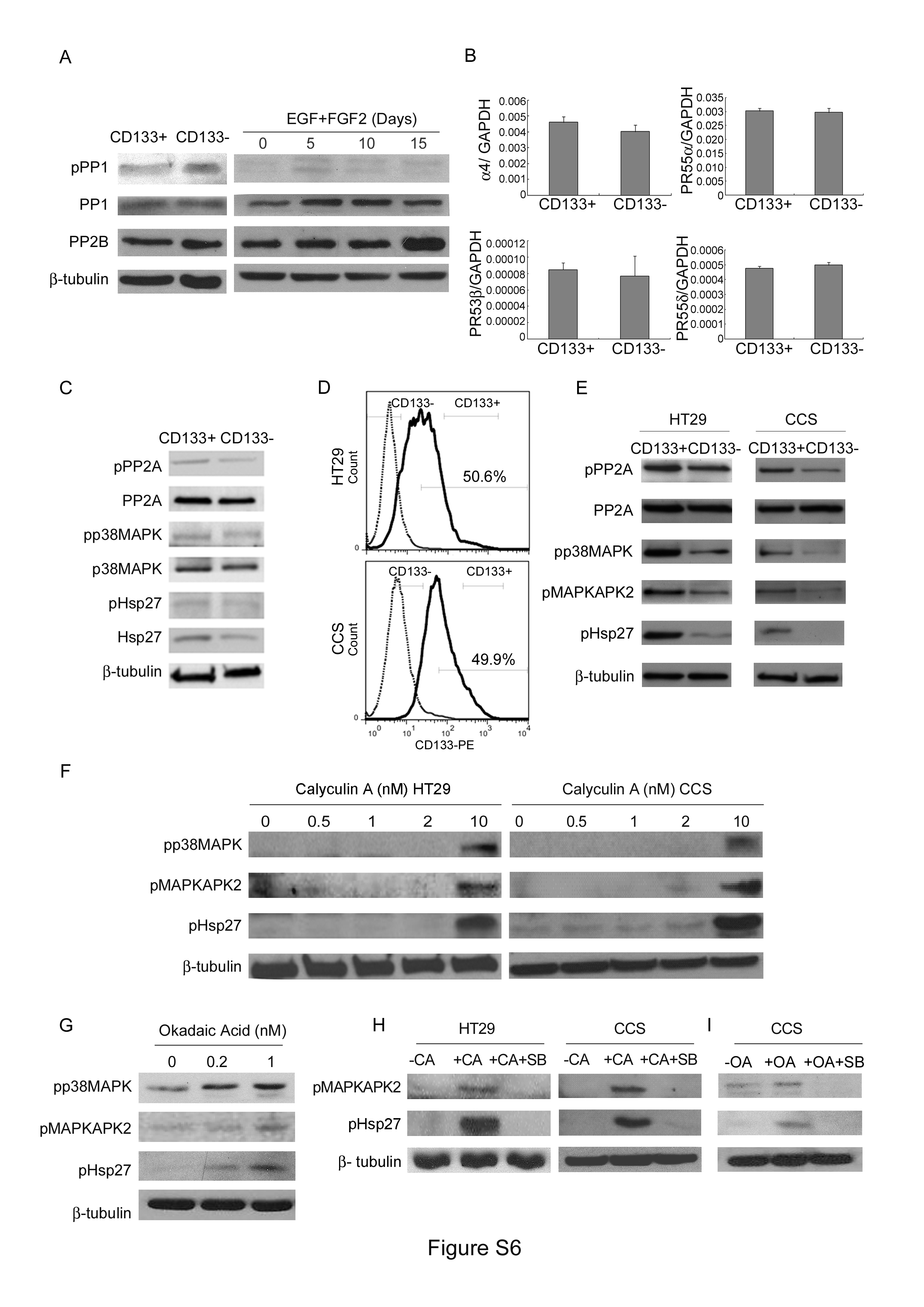

Supplement: Figure S6 — Decrease in PP2A increases phosphorylation of p38MAPK, pMAPKAPK2 and Hsp27 in enriched tumor initiating cells. (A left panel) Immunoblot analysis for HT-29 CD133+ and CD133− cells after MACS separation at day 4 of exposure to hypoxia and serum depletion. (A right panel) Immunoblots of CCS cells cultured under serum depletion in the presence of EGF (10 ng/mL) and FGF2 (10 ng/mL) (EGF+FGF2) for indicated time periods. (B) Quantitative RT-PCR for mRNA levels of HT-29 CD133+ and CD133− cells after MACS separation at day 4 of exposure to hypoxia and serum depletion. Bars indicate the expression ratio. (C) Immunoblots of HT29 CD133+ and CD133− cells under normal growth condition (FBS/Nor). (D, E) Flow cytometry and sorting for CD133+ and CD133− fractions in condition of EGF+FGF2. Dotted line represents isotype control. Ranges for sorting are shown in the upper region of the histogram. (E) Cell lysates were subjected for western blotting analysis. (F, G) HT-29 or CCS cells culture in the presence of Calyculin A (CA) and Okadaic acid (OA) at indicated concentrations or as a control, with DMSO, and immunoblot analysis was done at 30 min later. (H, I) HT-29 and CCS cells cultured in the presence of CA or OA with or without SB203580 at 50 µM (SB) or as a control, with DMSO, and immunoblot analysis was done at 30 min later. (TIF) [file pone.0049605.s006.tif]

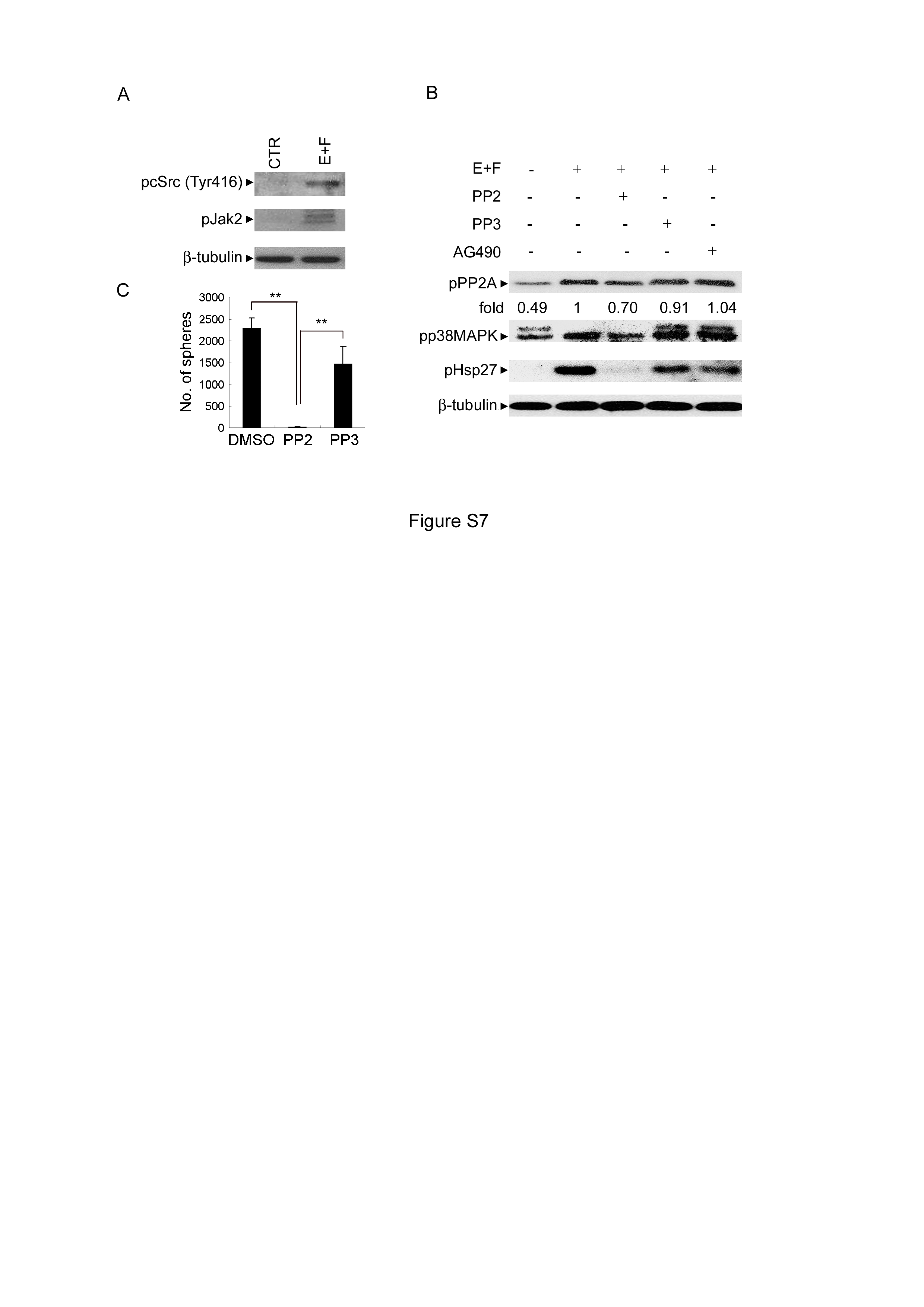

Supplement: Figure S7 — The involvement of c-Src activation in reducing PP2A activity and inducing sphere formation. (A) Immunoblots of CCS cells cultured in control growth medium or under serum depletion in the presence of EGF (10 ng/mL) and FGF2 (10 ng/mL) (EGF+FGF2) for 10 days. (B) Immunoblots of CCS cell grown under serum depletion in the presence of EGF (10 ng/mL) and FGF2 (10 ng/mL) (EGF+FGF2) for 15 days with DMSO (vehicle control), PP2 (10 µM), PP3 (10 µM) or AG490 (10 µM). Fold of pPP2A was normalized with the level of β-tubulin. (C) Spheres formed by CCS cell grown under serum depletion in the presence of EGF (10 ng/mL) and FGF2 (10 ng/mL) (EGF+FGF2) for 15 days with DMSO (vehicle control), PP2 (10 µM), and PP3 (10 µM) (n = 3). (*p<0.05 and **p<0.01 compared with DMSO as determined by the Student’s t test.). (TIF) [file pone.0049605.s007.tif]
